# Angular spectrum simulation of X-ray focusing by Fresnel zone plates

**DOI:** 10.1107/S090904951300263X

**Published:** 2013-03-12

**Authors:** Joan Vila-Comamala, Michael Wojcik, Ana Diaz, Manuel Guizar-Sicairos, Cameron M. Kewish, Steve Wang, Christian David

**Affiliations:** a Paul Scherrer Institut, 5232 Villigen PSI, Switzerland; b Argonne National Laboratory, Argonne, IL 60439, USA; c Synchrotron SOLEIL, Saint Aubin, BP-48, 91192 Gif-sur-Yvette, France

**Keywords:** diffractive X-ray optics, X-ray wavefield modeling, angular spectrum method, Fresnel zone plate stacking

## Abstract

A computing simulation routine to model any type of circularly symmetric diffractive X-ray element has been implemented. The wavefield transmitted beyond the diffractive structures is numerically computed by the angular spectrum propagation method to an arbitrary propagation distance. Cylindrical symmetry is exploited to reduce the computation and memory requirements while preserving the accuracy of the numerical calculation through a quasi-discrete Hankel transform algorithm, an approach described by Guizar-Sicairos & Gutierrez-Vega [*J. Opt. Soc. Am. A*, (2004 ▶), **21**, 53–58]. In particular, the code has been used to investigate the requirements for the stacking of two high-resolution Fresnel zone plates with an outermost zone width of 20 nm.

## Abstract 2

An efficient computing simulation routine has been implemented to model explicitly several types of Fresnel zone plate taking advantage of the circular symmetry. This code was used to evaluate an optimized approach for stacking of two high-resolution Fresnel zone plates.

## 1. Introduction   

Diffractive X-ray optics, and Fresnel zone plates (FZPs) in particular, are widely used optical elements for X-ray focusing and imaging (Sakdinawat & Attwood, 2010 ▶) with a broad range of applications in materials science (Zschech *et al.*, 2008 ▶; Nelson *et al.*, 2011 ▶, 2012 ▶; Wang *et al.*, 2012 ▶; Liu *et al.*, 2012 ▶; Vila-Comamala *et al.*, 2012 ▶) and biology (Larabell & Nugent, 2010 ▶; Schneider *et al.*, 2010 ▶; Stampanoni *et al.*, 2010 ▶; Andrews *et al.*, 2010 ▶; Mokso *et al.*, 2012 ▶; Chichon *et al.*, 2012 ▶). FZPs consist of circular diffraction gratings with radially increasing line density, which diffract and focus the incident X-ray beam into several foci, corresponding to different diffraction orders. The analytical theory describing FZPs in the extreme ultraviolet and X-ray regimes was first described by Kirz (1974 ▶) and further developed in several textbooks (see, for example, Michette, 1986 ▶; Attwood, 2000 ▶; Howells *et al.*, 2008 ▶). Nevertheless, these analytical descriptions mainly apply to conventional ideal binary FZPs and do not include other types of FZPs that have been proposed (Simpson & Michette, 1984 ▶; Jefimovs *et al.*, 2007 ▶) and are nowadays being used (Vila-Comamala *et al.*, 2011 ▶; David *et al.*, 2011 ▶). For such modified FZP geometries, numerical modeling is a suitable approach providing a theoretical insight into the details of how these devices perform.

Here, we have implemented a computer code to simulate the optical wavefield generated by FZPs employing the angular spectrum representation (Goodman, 1996 ▶; Novotny & Hecht, 2006 ▶). The computation requirements are reduced using the circular symmetry of FZPs, following the method described by Guizar-Sicairos & Gutierrez-Vega (2004 ▶). We illustrate the versatility of the code for modeling several FZP geometries and materials and for computing important parameters such as the diffraction efficiencies and the shapes of the focal spots. In addition, we use the routine to consider the case of two high-resolution FZPs (outermost zone width 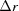 > 30 nm) stacked in the near-field (Maser *et al.*, 2002 ▶; Aristov *et al.*, 2007 ▶; Snigireva *et al.*, 2007 ▶; Feng *et al.*, 2007 ▶). In the hard X-ray regime, increasing the effective zone height by coupling two FZPs is a feasible solution to achieve higher diffraction efficiencies that may not be reachable by a single diffractive element when limitations of the FZP fabrication process exist. In the last section, we exemplify the application of our code to study an alternative stacking approach that relaxes the requirements for the experimental realisation of high-resolution FZP near-field stacking.

## 2. Methods   

In the X-ray regime, FZPs can have typical diameters as great as a few hundred micrometers and they are, at the same time, comprised of rings that can be as small as 10 nm at the outer region of the diffractive element. A numerical simulation that involves a full description and modeling of the FZP geometry with a precision of a few nanometers is computationally intensive. For example, using a sampling step of 0.5 nm to model a one-dimensional 100 µm-diameter FZP profile requires of the order of 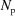 = 10^5^ points. A similar calculation using a two-dimensional model becomes significantly more challenging as it requires of the order of 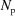 = 10^10^ points. Unless a reduced portion of the FZP is taken as input for two-dimensional calculation (Mastropietro *et al.*, 2011 ▶) or large computing facilities are used, the simulations must be restricted to a one-dimensional profile to avoid incurring currently unfeasible computer memory requirements.

**Figure 1:**
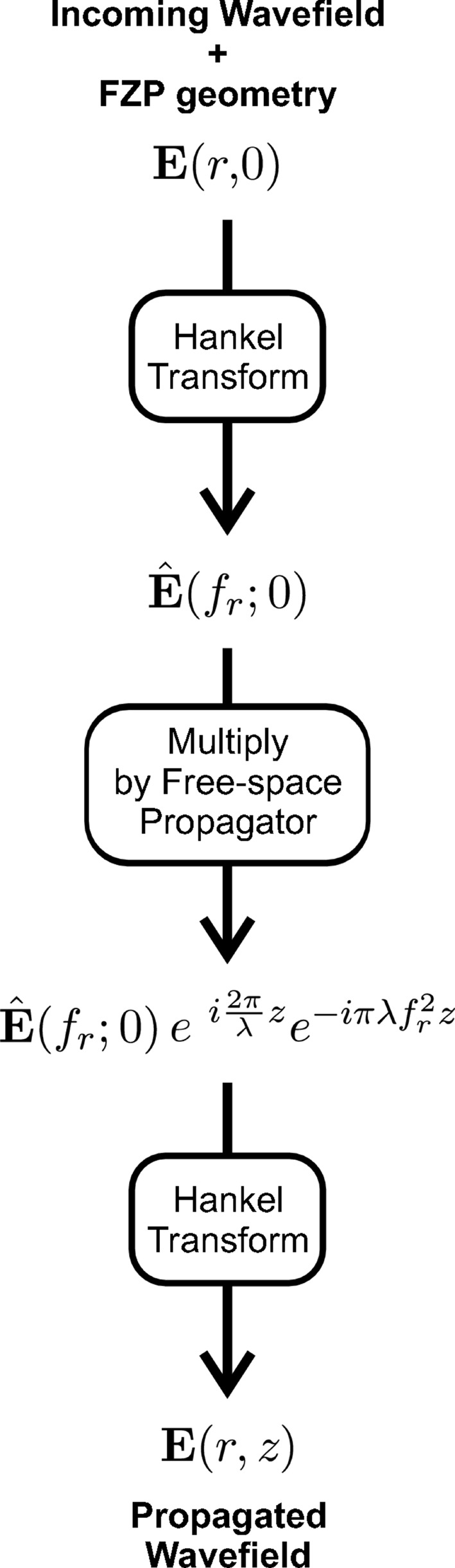
Scheme of the angular spectrum propagation method for a circularly symmetric optical wavefield, 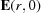. The angular spectrum of the initial wavefield, 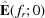, is calculated by a Hankel transform. Then, the propagated wavefield at a distance *z*, 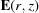, is obtained by multiplying 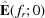 by the free-space propagator 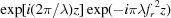 and applying a second Hankel transform.

In the past, computer simulations for the propagated optical wavefield produced by diffractive X-ray optics have been implemented by numerically solving the parabolic wave equation (Kopylov *et al.*, 1995 ▶; Kurokhtin & Popov, 2002 ▶). In this work we employ the angular spectrum method (Goodman, 1996 ▶; Novotny & Hecht, 2006 ▶), which is an exact solution to the scalar Helmholtz equation and is commonly used to simulate optical wavefield propagation. An initial circularly symmetric wavefield, **E**(*r*, *z* = 0), is assumed to be transversely coherent monochromatic radiation of wavelength λ. The circular symmetry around the optical *z*-axis simplifies the relation of a two-dimensional optical wavefield and its angular spectrum to a one-dimensional problem, since a two-dimensional Fourier transform can be expressed as a one-dimensional Hankel transform (Goodman, 1996 ▶). As schematically shown in the diagram in Fig. 1 ▶, the propagated wavefield at a distance *z*, 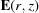, is obtained by multiplying the angular spectrum of the initial wavefield, 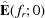, by the free-space propagator, 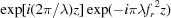, and applying a second Hankel transform. For simplicity, the expression of the free-space propagator is taken here in its paraxial approximation.^1^ A detailed summary of the involved mathematical expressions is explicitly given in Appendix *A* [App appa].

A fundamental step in this approach is the choice of a stable and accurate algorithm for the Hankel transform. Following earlier works, we applied a quasi-discrete Hankel transform (QDHT) routine (Yu *et al.*, 1998 ▶; Guizar-Sicairos & Gutierrez-Vega, 2004 ▶) to model diffractive X-ray optics because it gives a very high accuracy agreement with the exact analytical transforms and it requires neither the interpolation of the data points nor the use of extensive zero padding. Unlike other Hankel transform algorithms such as the one used by Vila-Comamala *et al.* (2011 ▶), the QDHT is energy preserving by construction and the original function is retrieved after two successive applications of the Hankel transform with a precision comparable with the fast Fourier transform routines. For the zero-order QDHT, the function to be transformed is sampled at points proportional to the roots of the zero-order Bessel function of the first kind. The sampling points approximate a regular sampling grid for large *r* values and the QDHT computation is expressed as the product of a transformation matrix and the input wavefield vector. When the initial wavefield was sampled with a large number of points, 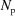 > 10^4^, the QDHT matrix required an allocation of memory larger than available in our computing system. Hence, the matrix–vector multiplication was divided into smaller tasks of vector–vector products. Since this problem was inherently parallel, we were able to take advantage of multi-core processors to speed up the computations.

The initial wavefield was modeled assuming a plane wave of unit amplitude incident on the FZP and by introducing the effects of the FZP geometry, height and material using the tabulated refractive index values.^2^ Considering the thin element approximation, the optical wavefield exiting the FZP is calculated as the product of the incoming plane wavefield and the complex-valued amplitude transmittance function of the diffractive optical element, which includes both absorption and phase-shifting effects. For example, for an ordinary binary FZP made of a total of *N* zones, material height *h*, refraction index *n* = 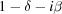, diameter *D* and focal length 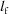, the optical wavefield exiting the diffractive optical element can be written as
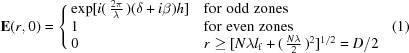
where, for odd zones, 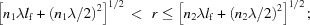
for even zones 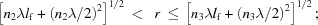
and 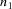 = 0, 2, 4,…*N*; 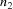 = 1, 3, 5,…*N*; 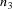 = 2, 4, 6,…*N*. The wavefield propagation routine was implemented in MATLAB. The discretization of the initial wavefield in equation (1) was typically performed with a total number of points, 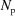, ranging from 10000 to 80000 to ensure a fine sampling of the FZP profile at the outer region of the structure. The maximum radius of the simulated real space was taken typically as four times the radius of the FZP to ensure fine sampling in reciprocal space. The simulations and calculations shown in the next sections were computed using an Intel Core i7-2600 3.40 GHz CPU and 12 GB of RAM. The parallelization of the QDHT algorithm using eight CPU threads reduces the computation time by almost a factor of seven. The typical time to calculate a single QDHT and the wavefield at a given *z* with a discretization of 20000 points was 30 s. The computing time increased to 8 min for 80000 points, scaling with the square of the number of points as expected.

## 3. Results and discussion   

The implemented computer routines were used to study the focal spots shapes and diffraction efficiencies of different types of FZPs, and to investigate the behavior of a two-stacked FZP system. Different FZP types were implemented by using their amplitude transmittance functions similar to the expressions in equation (1) for a conventional binary FZP.

### 3.1. Studying several types of FZP geometries   

**Figure 2:**
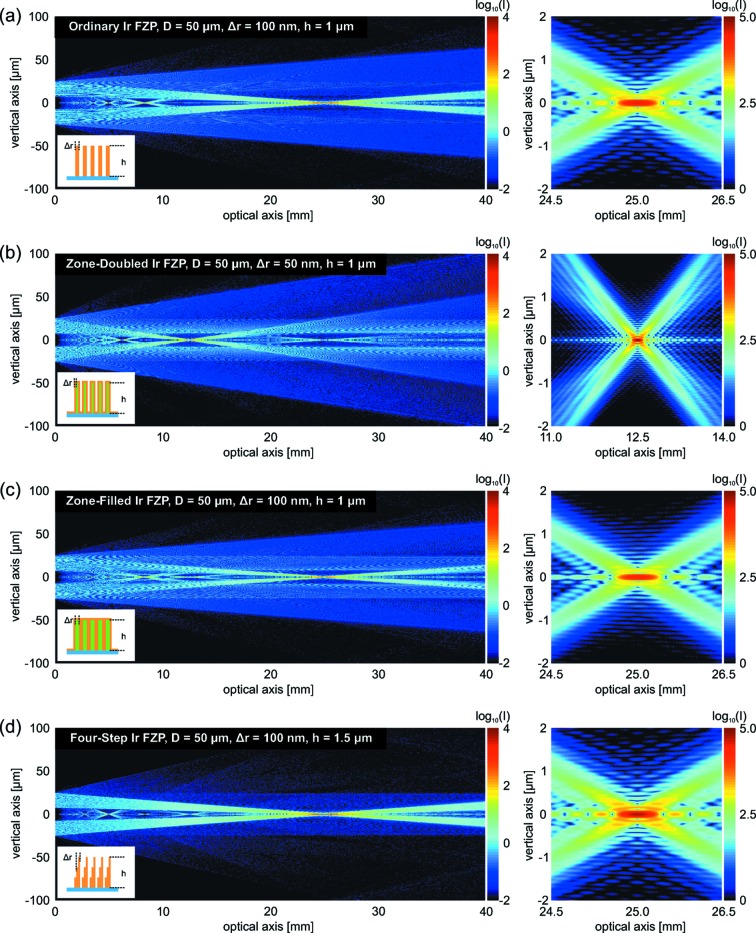
Intensity of the propagated wavefield created by four different types of FZPs with a diameter of 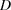 = 50 µm. (*a*) Ordinary, (*b*) zone-doubled, (*c*) zone-filled and (*d*) four-level staircase FZP geometries are considered. A photon energy of 6.2 keV, *i.e.* wavelength of 0.2 nm, is assumed.

Fig. 2 ▶ shows the intensity of wavefields produced by four different types of FZPs, as they propagate along the *z*-axis to their focal position and further. The calculations were performed considering a photon energy of 6.2 keV (wavelength λ = 0.2 nm). The FZP structures are made of iridium (atomic number *Z* = 77, mass density ρ = 22.56 g cm^−3^) and have a diameter of *D* = 50 µm. A completely opaque central stop of diameter 15 µm blocks the central part of the incoming radiation. Fig. 2(*a*) ▶ shows the propagated wavefield of an ordinary FZP with an outermost zone width of 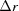 = 100 nm. A zone height of *h* = 1 µm was chosen to give an almost optimal diffraction efficiency for the selected X-ray energy. As expected, the focal spot is located at a distance 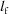 = 25 mm downstream of the FZP. The higher-diffraction-order focal spots can also be observed at the expected axial positions. Fig. 2(*b*) ▶ demonstrates the performance of a zone-doubled FZP (Vila-Comamala *et al.*, 2011 ▶) with an effective outermost zone width of 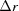 = 50 nm. In this case the FZP structure is made by a combination of iridium and a silicon-oxide-like inorganic resist. The focal spot is located at 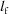 = 12.5 mm and it is twice as small as that of the ordinary FZP. The next simulated diffractive optical element in Fig. 2(*c*) ▶ is a zone-filled FZP made of iridium and diamond (atomic number *Z* = 6, mass density ρ = 3.52 g cm^−3^). Such FZPs have been used to focus X-ray free-electron laser (XFEL) radiation (David *et al.*, 2011 ▶), as the combination of diamond and iridium proves capable of withstanding the large heat load of the XFEL radiation pulses and provides a reasonable diffraction efficiency. The calculation provides confirmation that the quality and shape of the focus remains unchanged from that created by an ordinary FZP, and that only the diffraction efficiency is decreased. Finally, Fig. 2(*d*) ▶ shows the intensity of the wavefield generated by a FZP made of a four-level staircase profile. This diffractive optical element approximates the ideal shape of a Fresnel lens (Di Fabrizio *et al.*, 1999 ▶; Nohammer *et al.*, 2003 ▶) necessary to maximize the diffraction efficiency of the first-order focus. In this case there is much more intensity diffracted to the first-order focal spot and there is a substantial reduction of the intensity of the first divergent diffraction order in comparison with an ordinary FZP. The height of the structure was chosen to be *h* = 1.5 µm, which is an almost ideal value for 6.2 keV photon energy. The large intensity maps in Fig. 2 ▶ were calculated at 400 optical axis positions and sampling the FZP profiles with 25000 points. The computing time to obtain each of them was approximately 5 h.

**Figure 3:**
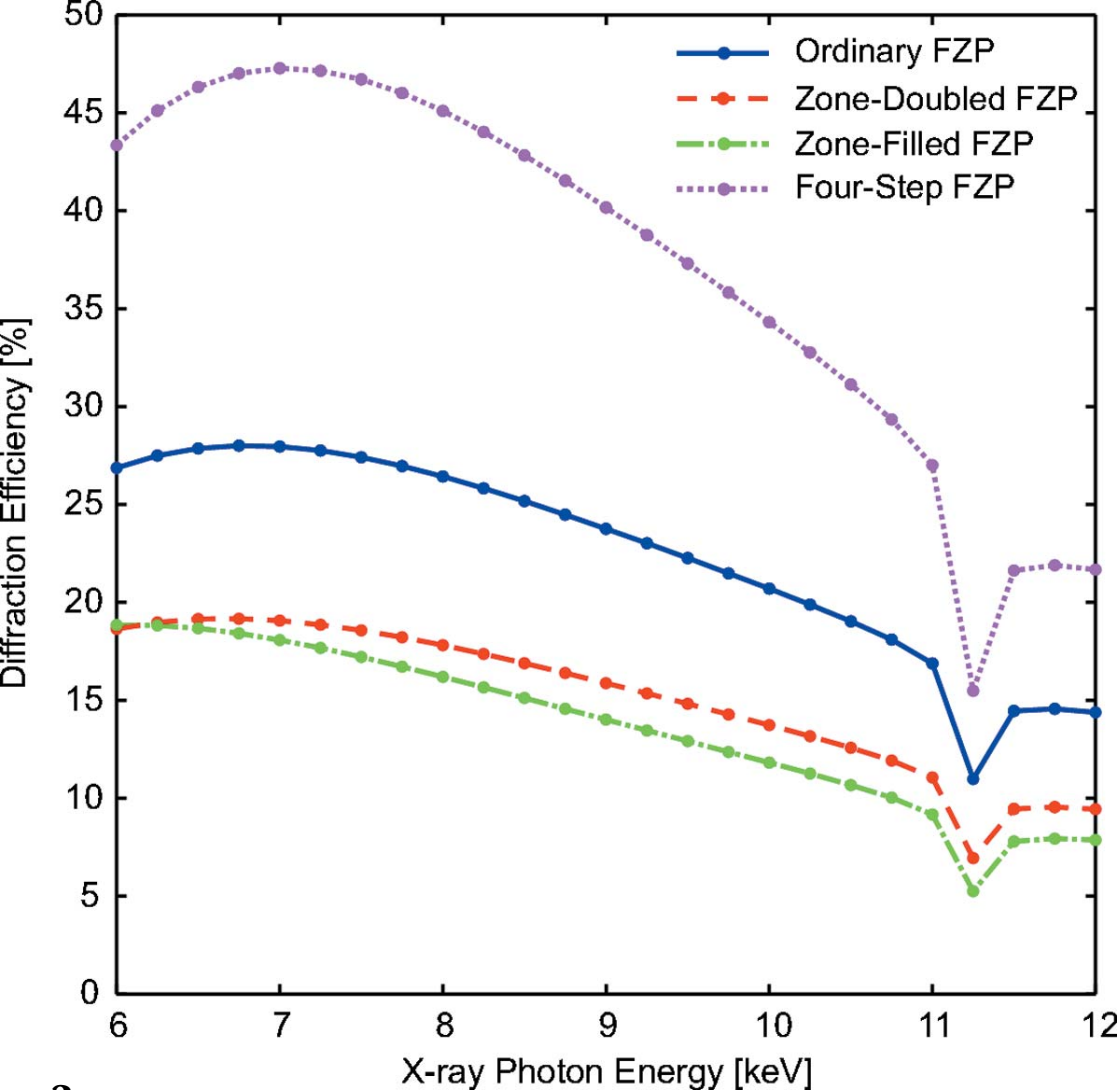
Calculated diffraction efficiencies for four iridium FZP types considering an X-ray energy range from 6 to 12 keV. The diffraction efficiencies of the zone-doubled and zone-filled FZPs are about 35% lower than those from ordinary FZPs. The four-step staircase FZP displays a remarkable diffraction efficiency increase. The iridium *L* absorption edge generates an abrupt decrease of the diffraction efficiency at an energy of 11.2 keV.

The simulation code can also be used to estimate the diffraction efficiency of the structures by integrating the wavefield intensity at the focal plane. Fig. 3 ▶ shows the calculated diffraction efficiency for the four types of FZP patterns as a function of the photon energy in the range from 6 keV to 12 keV. The diffraction efficiency of the ordinary FZP matches the curve that can be calculated from analytical expressions (Kirz, 1974 ▶). The diffraction efficiencies of the zone-doubled and zone-filled FZPs are about 35% lower in comparison with an ordinary FZP. On the other hand, the efficiency of the four-level staircase FZP is substantially higher, as expected. The staircase profile breaks the symmetry between the convergent (positive) and divergent (negative) diffraction orders in ordinary FZPs. The increase of diffraction efficiency occurs at the expense of substantially reducing the intensity of the zero and first divergent diffractive orders.

### 3.2. Stacking of FZPs   

**Figure 4:**
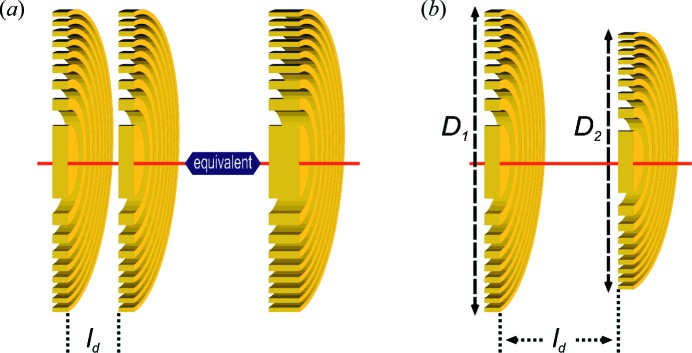
Stacking of two FZPs. (*a*) Two identical FZPs separated by a very short distance 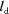 can be stacked in the near-field to obtain an equivalent thicker structure and to achieve a substantial increase in diffraction efficiency. (*b*) The close proximity requirement on the two stacked FZPs can be relaxed by adjusting the diameter, 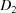, of the second diffractive optical element.

Another interesting problem that can be studied by this wavefield propagation computer routine is the case of two FZPs stacked in the near-field (Maser *et al.*, 2002 ▶; Aristov *et al.*, 2007 ▶; Snigireva *et al.*, 2007 ▶; Feng *et al.*, 2007 ▶). Especially in the hard X-ray regime, the use of a FZP pair is an interesting approach to achieve higher diffraction efficiency and overcome the limitations on the production of high-aspect-ratio diffractive optical elements imposed by the nanofabrication technology. As schematically shown in Fig. 4(*a*) ▶, two identical FZPs can be aligned in close proximity to obtain an element equivalent to a thicker FZP with increased diffraction efficiency. In this case the calculation is performed by propagating the wavefield exiting the first FZP to the position of the second FZP, by multiplying by its amplitude transmittance function and propagating further the resulting wavefield to the focal plane position. The thin element approximation is assumed for the effect of both FZPs on the propagating wavefield.

The condition for near-field stacking of two FZPs can be derived from geometric considerations, as the product of separation distance, 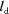, and the angle of lateral deviation, 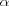 = 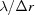, of the wavefield produced by the FZP geometry should remain smaller than half of the outermost zone width of the FZP, 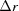; that is,
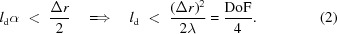
The depth of focus (DoF) is defined here as usual, 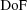 = 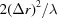. An additional condition for the near-field stacking not considered here is that the two FZPs need to be aligned laterally and angularly to ensure that they work as a single element. In former works, the required zone placement accuracy in a FZP was shown to be one-third of the outermost zone width (Michette, 1986 ▶).

**Figure 5:**
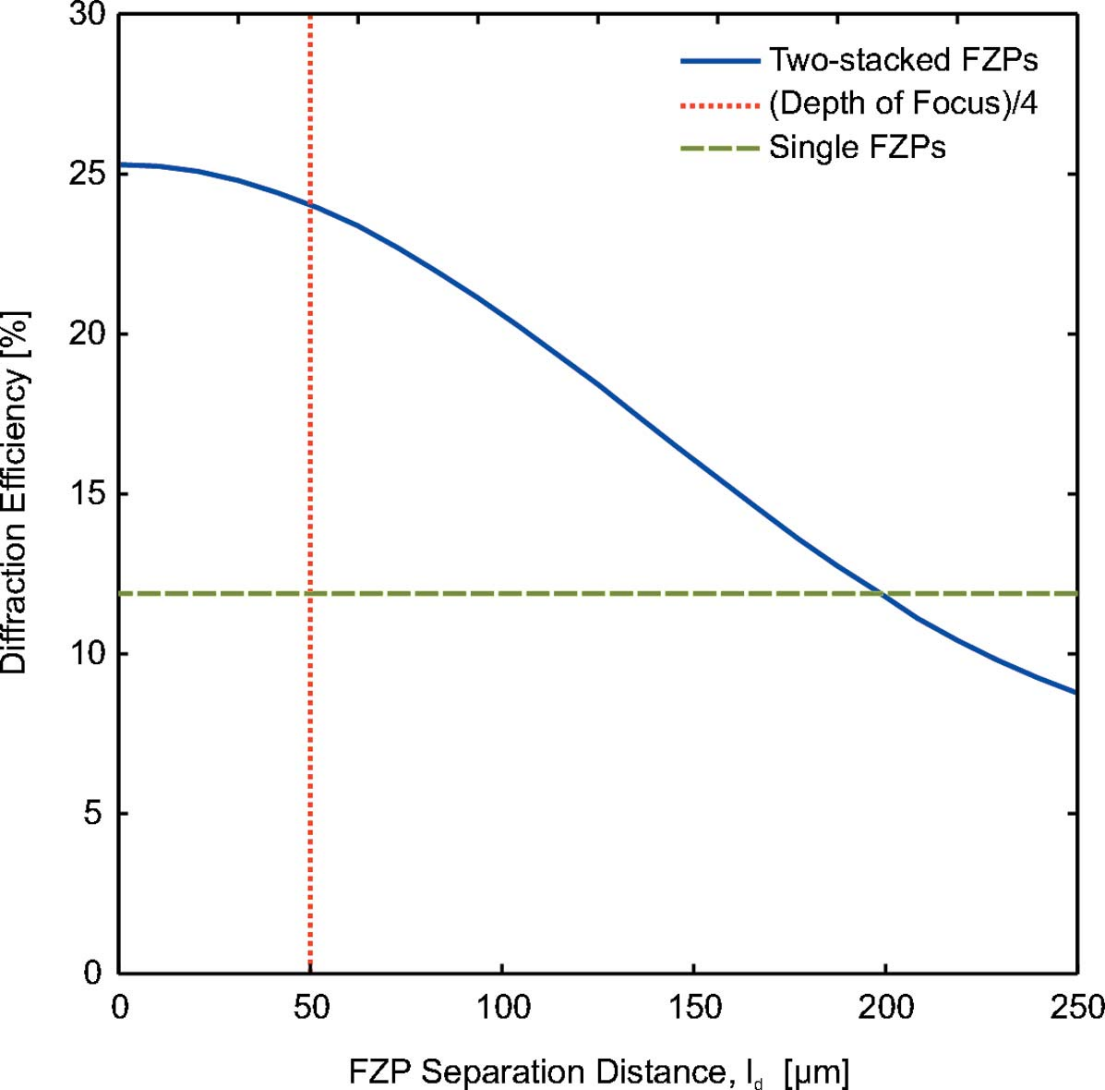
Calculated diffraction efficiency of two identical stacked gold FZPs with an outermost zone width of 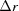 = 100 nm, a zone height of 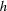 = 500 nm each and a photon energy of 6.2 keV. As the separation distance 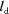 increases, the diffraction efficiency of the two stacked FZPs decreases. The diffraction efficiency of a single ordinary FZP with the same parameters is shown for comparison.

In the first place, we modeled two FZPs made of gold (atomic number *Z* = 79, mass density 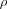 = 19.30 g cm^−3^) with a diameter of 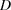 = 100 µm, an outermost zone width of 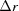 = 100 nm and a zone height of 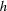 = 500 nm for a photon energy of 6.2 keV. Fig. 5 ▶ shows the diffraction efficiency of the FZP doublet as a function of the separation distance, 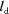. The plot demonstrates that for separation distances below 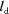 = 25 µm there is a negligible effect on the paired system. Up to distances of about one-quarter of the depth of focus, the diffraction efficiency slowly decreases. This result is consistent with the near-field stacking condition in equation (2).

Thus, the stacking of these two FZPs with an outermost zone width of 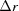 = 100 nm can be accomplished at a relatively large separation distance of 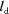 = 50 µm between the two elements. Such a separation distance can be achieved by producing the two diffractive optical elements in two independent chips and placing them together using some mechanical mounting that allows for the control and positioning of the two elements with an accuracy of a few nanometers in the directions perpendicular to the X-ray beam and with a precision of a few micrometers in the optical axis direction. However, when trying the same approach for a high-resolution FZP with an outermost zone width of 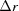 = 25 nm, the near-field condition of *d* 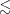 3 µm is significantly more challenging to realise by mechanically pushing the two FZPs chips together. An extremely high accuracy in the machining of the mounting holders is required to achieve this condition and any imperfection or particle between the FZP chips can dramatically affect the performance of the pair.

**Figure 6:**
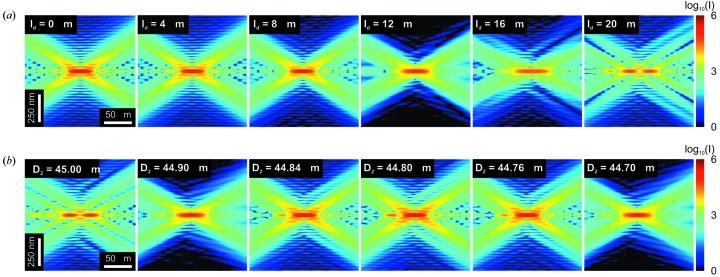
Simulated wavefield intensity in the vicinity of the focal spots created by two stacked zone-doubled FZPs with an outermost zone width of 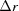 = 25 nm. (*a*) When two FZPs of identical diameter, 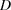 = 45 µm, are stacked, the separation distance required for an acceptable focal spot shape is below 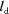 = 10 µm. (*b*) The separation distance can be relaxed by adjusting the diameter, 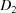, of the second FZP. For a separation distance of 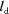 = 25 µm an optimal focus profile is recovered for a diameter 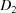 = 44.8 µm.

**Figure 8:**
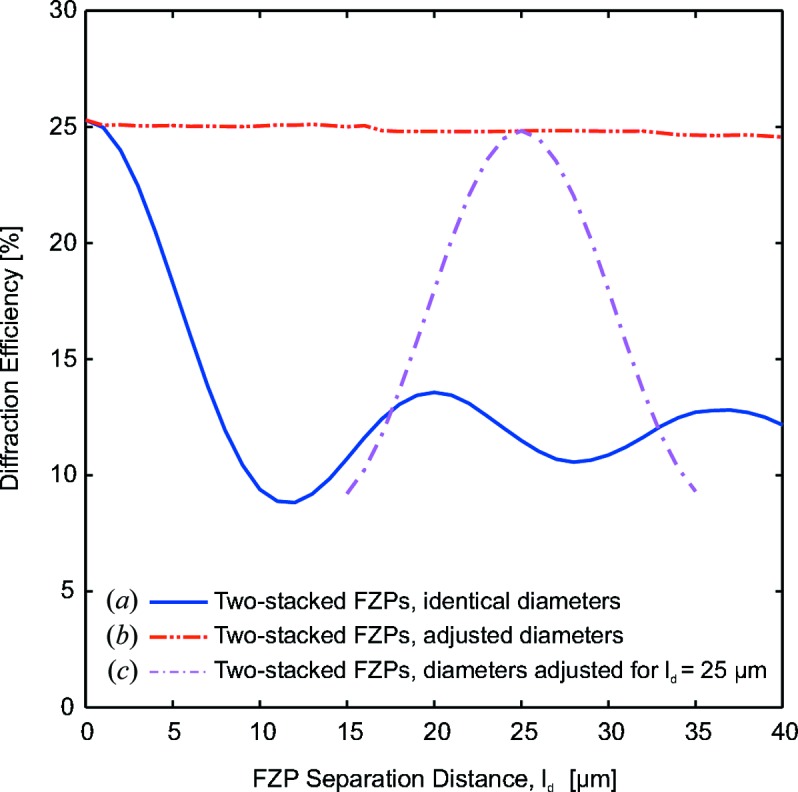
Calculated diffraction efficiency for the two stacked high-resolution ordinary gold FZPs with an outermost zone width of 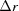 = 20 nm and photon energy of 6.2 keV. (*a*) The diffraction efficiency decreases rapidly as a function of the separation distance 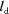 when the two stacked FZPs have identical diameters. (*b*) The diffraction efficiency is kept constant when the diameter of the second FZP is optimized for every separation distance. (*c*) The diffractive efficiency for an experimentally realisable system in which the second FZP has a diameter adjusted for a separation distance of 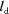 = 25 µm.

To investigate the stacking of high-resolution FZPs, Figs. 6(*a*) ▶ and 7(*a*) ▶ show the propagated wavefields at the vicinity of the focal spot for two stacked zone-doubled FZPs with a diameter of 45 µm and an outermost zone width of 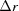 = 25 nm as a function of the separation distance, 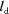, between the two FZPs. Besides providing the diffraction efficiency, the calculation demonstrates that the shape of the focal spot is affected when the separation is larger than 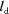 = 10 µm. On the other hand, as shown schematically in Fig. 4(*b*) ▶, the separation distance between the FZP can be relaxed by appropriately adjusting the diameter of the second FZP so that the first-order foci of both elements coincide along the optical axis. Figs. 6(*b*) ▶ and 7(*b*) ▶ show the propagated wavefields at the vicinity of the focal spot for two stacked zone-doubled FZPs with an outermost zone width of 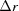 = 25 nm which are separated by a distance of 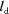 = 25 µm. The diameter of the first is chosen to be 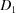 = 45 µm while the diameter of the second element, 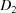, is varied in small steps. When the two diameters are identical, the focal spot is deformed and split in two along the optical axis. However, when the diameter of the second elements is taken as 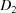 = 44.8 µm, the shape of the focal spot is recovered. The diameter, 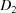, is such that
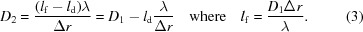
Finally, Fig. 8 ▶ shows the diffraction efficiency as a function of the separation distance, 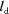, for the stacking of two high-resolution FZPs. Now, we consider two conventional FZPs made of gold with an outermost zone width of 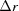 = 20 nm and diameter of 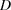 = 25 µm. Three different situations are examined. In the case of two FZPs of identical diameter, the diffraction efficiency decreases steeply as a function of the separation distance and values of 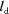 < 5 µm are necessary to avoid a significant decrease in the diffraction efficiency. The second scenario considers two FZPs for which the diameter of the second FZP is adjusted according to equation (3). The resulting flat curve demonstrates that for any separation distance a second lens can be made with a suitable diameter. A third situation considers the stacking of two FZPs for which the diameter of the second element is chosen for a separation distance of 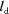 = 25 µm. The curve demonstrates that, in addition to being able to relax the separation distance for optimal performance, the range of tolerance for the relative position error is actually doubled in comparison with the stacking of two identical FZPs for a near-field condition separation distance because one can err either in the positive or negative optical axis direction. From an experimental point of view, this is a very relevant result as it relaxes the otherwise strict requirements for the stacking of two high-resolution FZPs in the near-field.

## 4. Conclusions   

In this work we have implemented a computer routine based on the angular spectrum representation to simulate the propagation of the optical wavefield produced by circularly symmetric X-ray diffractive optical elements. We showed that the routine can be used to simulate the performance of several types of FZPs. In the future, the calculations will be extended to consider cases including errors in the FZPs patterns that arise from the fabrication tolerances involved in the production of X-ray FZPs and the dynamical diffraction phenomena caused by the propagation of the wavefield through the FZP (Pfeiffer *et al.*, 2006 ▶). We have also applied the computer routines to investigate the stacking of two high-resolution FZPs. The calculations demonstrate that the stringent conditions imposed on the separation distance between the two elements for near-field stacking can be relaxed by adjusting the diameter of the FZP, thus enabling a feasible future experimental realisation. By generalizing this result to systems of three or more FZPs, one could substantially improve the throughput of FZP-based X-ray microscopes and achieve higher diffraction efficiencies for hard X-ray energies (>20.0 keV) for which FZPs are currently ineffective.

## Figures and Tables

**Figure 7:**
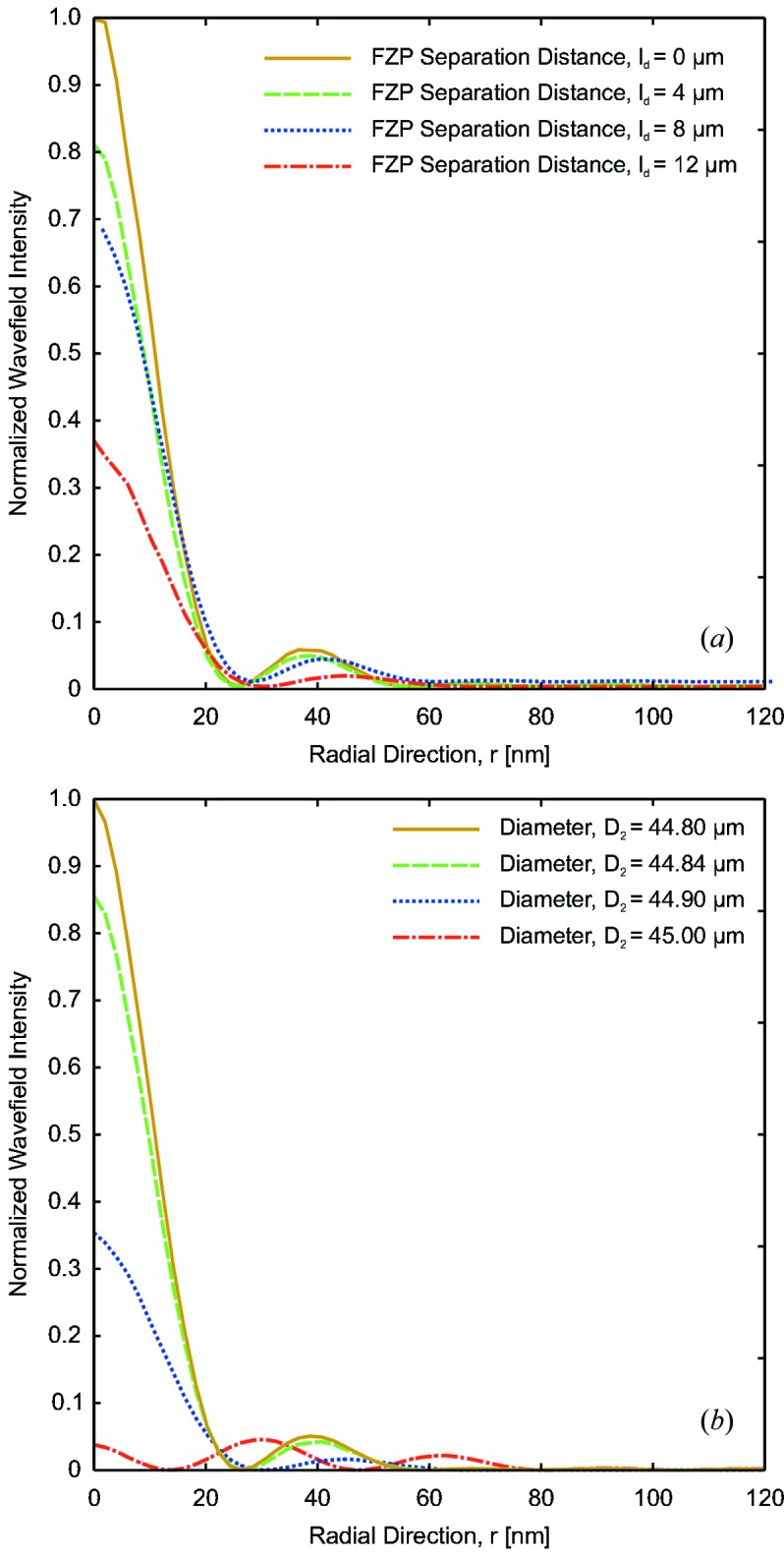
Normalized intensity of the focal spots created by two stacked zone-doubled FZPs with an outermost zone width of 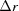 = 25 nm. (*a*) With two FZPs of identical diameter, 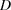 = 45 µm, the focal spot is distorted by increasing their separation distance, 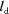. (*b*) For a chosen separation distance of 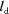 = 25 µm, the optimal focal spot is recovered when the diameter of the second FZP is adjusted to 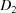 = 44.8 µm.

## Footnotes

1 This condition is almost always met for diffractive optics in the X-ray regime, since the involved numerical apertures are usually very small; .

2 Center for X-ray Optics; *X-ray Interactions With Matter*, http://www.cxro.lbl.gov.

## Appendix A Angular spectrum representation

The angular spectrum representation (Goodman, 1996 ▶; Novotny & Hecht, 2006 ▶) is a mathematical approach that describes the propagation of scalar electromagnetic wavefields in homogeneous media and is an exact solution of the scalar Helmholtz equation. Although this is a widely used procedure for wavefield propagation, for completeness here we rewrite the mathematical equations usually expressed in Cartesian coordinates using the cylindrical coordinates. Following the notation in Goodman (1996 ▶), we define the angular spectrum, or Fourier spectrum,  of an optical wavefield  at a plane *z* as

To take advantage of the symmetry of the problem we introduce the cylindrical coordinates  and ,

and, taking a circular symmetric wavefield  =  independent of θ, equation (4) can be expressed in terms of the zero-order Hankel transform (Goodman, 1996 ▶),

Thus, in the particular case of two-dimensional circular symmetric functions, their two-dimensional Fourier transform can be expressed as a one-dimensional Hankel transform. Similarly, the inverse relation can be written as

by taking into account that there is no difference between the Hankel transform and the inverse Hankel transform. Upon the wavefield propagation, the angular spectrum  evolves along the *z*-axis according to

In some cases it may be desirable and appropriate to take the paraxial (small-angle) approximation,

Finally, the propagated optical wavefield  can be expressed as
